# Is There a Link between Exertional Heat Stroke and Susceptibility to Malignant Hyperthermia?

**DOI:** 10.1371/journal.pone.0135496

**Published:** 2015-08-10

**Authors:** Emmanuel Sagui, Coline Montigon, Amandine Abriat, Arnaud Jouvion, Sandrine Duron-Martinaud, Frédéric Canini, Fabien Zagnoli, David Bendahan, Dominique Figarella-Branger, Michel Brégigeon, Christian Brosset

**Affiliations:** 1 French Military Hospital Laveran, Marseille, France; 2 Val de Grâce Military School, Paris, France; 3 Unité mixte de recherche 7291, laboratoire de neurosciences cognitives, Aix Marseille University, Marseille, France; 4 French Fire Brigade Health Service, Courbevoie, France; 5 Armed Forces Epidemiological and Public Health Center, Marseille, France; 6 Armed Forces Biomedical Research Institute, Brétigny/Orge, France; 7 French Military Hospital Clermont-Tonnerre, Brest, France; 8 Unité mixte de recherché 7339, Center for Magnetic Resonance in Biology and Medicine, Aix Marseille University, Marseille, France; 9 Service d’anatomie pathologique et de neuropathologie, Timone hospital, assistance publique/hôpitaux de Marseille, Marseille, France; West Virginia University School of Medicine, UNITED STATES

## Abstract

**Objective:**

The identification of a predisposition toward malignant hyperthermia (MH) as a risk factor for exertional heat stroke (EHS) remains a matter of debate. Such a predisposition indicates a causal role for MH susceptibility (MHS) after EHS in certain national recommendations and has led to the use of an *in vitro* contracture test (IVCT) to identify the MHS trait in selected or unselected EHS patients. The aim of this study was to determine whether the MHS trait is associated with EHS.

**Methods:**

EHS subjects in the French Armed Forces were routinely examined for MHS after experiencing an EHS episode. This retrospective study compared the features of IVCT-diagnosed MHS (iMHS) EHS subjects with those of MH-normal EHS patients and MH patients during the 2004–2010 period. MHS status was assessed using the European protocol.

**Results:**

During the study period, 466 subjects (median age 25 years; 31 women) underwent MHS status investigation following an EHS episode. None of the subjects reported previous MH events. An IVCT was performed in 454 cases and was diagnostic of MHS in 45.6% of the study population, of MH susceptibility to halothane in 18.5%, of MH susceptibility to caffeine in 9.9%, and of MH susceptibility to halothane and caffeine in 17.2%. There were no differences in the clinical features, biological features or outcomes of iMHS EHS subjects compared with those of MH-normal or caffeine or halothane MHS subjects without known prior EHS episode. The recurrence rate was 12.7% and was not associated with MH status or any clinical or biological features. iMHS EHS patients exhibited a significantly less informative IVCT response than MH patients.

**Conclusions:**

The unexpected high prevalence of the MHS trait after EHS suggested a latent disturbance of calcium homeostasis that accounted for the positive IVCT results. This study did not determine whether EHS patients have an increased risk of MH, and it could not determine whether MH susceptibility is a risk factor for EHS.

## Introduction

Malignant hyperthermia (MH) is a hypermetabolic reaction triggered by volatile anesthetics (except nitrous oxide) and succinylcholine [1]. MH is characterized by the uncontrolled release of intracellular calcium, generally in connection with mutations in the gene for either type 1 ryanodine receptor (RYR1), a calcium channel in the sarcoplasmic reticulum membrane responsible for the release of calcium during muscle contractions, or CACNA1S, encoding the α_1_ subunit of a Ca_v_1.1, a calcium channel located within the t-tubule membrane [2,3]. The main clinical features of MH include muscle rigidity, rhabdomyolysis, and severe hyperthermia. Susceptibility to MH is diagnosed by the *in vitro* contracture test (IVCT), an invasive procedure in which muscle fibers are exposed to halothane and caffeine according to two standardized protocols from the European MH Group and the North American MH Group [4,5].

Exertional heat stroke (EHS) is clinically defined as hyperthermia over 40°C, encephalopathy, and symptoms ranging from mild confusion to coma. EHS occurs during or immediately after strenuous physical activity in young, previously healthy subjects. EHS may be complicated by multi-organ failure, including acute renal failure (ARF) and hepatic failure, and may result in death in the absence of appropriate treatment [6].

In the late 1970s, Wingard et al. hypothesized that due to the clinical and biological similarities between EHS and MH, such as hyperthermia and rhabdomyolysis, EHS may be related to a latent skeletal muscle disorder triggered by strenuous exercise [7]. Furthermore, porcine MH, an animal model for human MH, was shown to be triggered by hyperthermia, which is also considered to be the trigger for EHS [8]. This link was substantiated by the description of EHS in patients with familial or personal histories of MH [9,10]. MH susceptibility has been viewed as a risk factor for EHS, and some authors have suggested that EHS subjects should be investigated by MH susceptibility screening [11–13]. A decade ago, Muldoon et al. suggested that individuals who collapse from exertional heat illness should be screened for MH. This raises two important questions: are individuals who have suffered from MH at risk for EHS, and are EHS subjects at risk for MH [12]?

However, establishing MH susceptibility as a risk factor for EHS implies that subjects are more likely to present EHS when they are MH-susceptible. Such studies would be difficult to perform because they would require an IVCT to determine the MH status prior to the onset of EHS; however, IVCT is an invasive and ethically unacceptable procedure among asymptomatic patients. Thus, the use of indirect data may present a solution.

In the French Armed Forces, a pilot study identified susceptibility to MH in 11 of 45 EHS subjects [14]. From 2005 to 2010, the incidence rate of EHS ranged from 18.3 to 36 per 100,000 person-years [15]. The use of the “person-years” as denominator is intended to take into account the different time contributions of the subjects. It refers to the amount of time a subject contributes to a study. For example, a subject who would be surveyed for 6 months will contribute to 0.5 person-years. Because of the presumed link between the two conditions and the high prevalence of EHS, all French military subjects with prior EHS histories underwent IVCT to assess their MH susceptibility [16]. The objective of this screening was to remove EHS subjects with the MH susceptibility trait from conditions that might elicit another EHS episode.

To evaluate the link between EHS and the MH susceptibility trait, we recorded data from subjects with prior EHS who were subsequently tested for MH susceptibility between 2004 and 2010.

## Methods

This study was approved by the Marseilles II Ethics Committee, France. All subjects provided an informed written consent statement prior to the study that included permission for muscle biopsies. All subjects were investigated in the Neurological Service of the Military Teaching Hospital Laveran, which operates as a national clinical reference center for EHS among the military population. First, a retrospective study was performed among French military subjects with prior EHS who were investigated for MH susceptibility between 2004 and 2010. Medical charts were individually reviewed, and demographic, historical, clinical and biological data were extracted. Due to the lack of a specific recording log at the time of the investigations, some clinical and biological data were missing for a few subjects. Second, subjects who had been investigated between 2008 and 2010 were contacted between April to July 2012 to assess the clinical features and recurrence rate of EHS via a semi-structured telephone conversation. A questionnaire was sent to the subjects for whom no telephone conversation was recorded. Third, quantitative IVCT results of the iMHS EHS subjects investigated between 2008 and 2010 were compared with the IVCT results for MH patients. All IVCT examinations for EHS and MH patients were performed in the same laboratory (a European MH group investigation unit; CNRS CRMBM UMR 7339, Aix Marseille University, 13385 Marseille, France). Due to the low prevalence of MH, all but one of the patients who were investigated from 2003 to 2011 were included.

EHS was defined in accordance with French military regulations [17], *i*.*e*., (i) the onset of central nervous system (CNS) impairment during or after intense and prolonged exercise and (ii) hyperthermia with a body core temperature (Tco) exceeding 39°C. Tco was measured in the field either rectally or by ear in all subjects. ARF was defined as a serum creatinine level greater than 170 μmol/l coupled with above-normal urea levels. Hepatocellular insufficiency was defined by cytolysis with a prothrombin ratio below 70% of normal. EHS recurrence was defined as the onset of another EHS using the same criteria.

IVCT status was determined at least three months after EHS according to most recommendations of the European protocol [4]. A positive contracture test was considered if the 2 mN threshold value for muscle tension was attained at concentrations of less than or equal to 2 mmol/l caffeine and 2% halothane. The IVCT results were classified as MH susceptible (MHS) if the results of halothane, caffeine or both of them were positive, MH susceptible for halothane (MHSh) if the IVCT results were positive for halothane, MH susceptible for caffeine (MHSc) if the IVCT results were positive for caffeine and MH normal (MHN) when neither test was positive. Muscle biopsies were performed in the *biceps brachialis* muscle rather than the *vastus lateralis* muscle because the recovery of *biceps brachialis* muscle function was more rapid and the IVCT results were not dependent on the muscle used for the biopsy, as shown previously [18].

Data were recorded using Epi info version 3.5.3 (CDC, Atlanta, GA, USA) and analyzed using SAS version 9.2 (SAS Inc., Cary, NC, USA) and Stata version 11 (Statacorp, College Station, TX, USA). Discrete variables were compared using the Chi squared test or Fisher’s exact test when required. Continuous variables with a normal distribution were compared using Student’s t-test; these data are presented as the mean±the standard deviation. Results with a non-normal distribution were compared using the Wilcoxon-Mann-Whitney test; these data are presented as the median and 25–75% interquartile interval (25%-75%). Logistic regression was performed when the effect of candidate variables had a p-value <0.20. A backward stepwise selection procedure was applied to retain significant independent variables with p-values <0.05. The Hosmer and Lemeshow test was used to assess the goodness of the fit of the model. A p-value <0.05 was considered significant.

## Results

From 2004 to 2010, 466 subjects were examined following an EHS episode (median age 25 years (22–29); 31 women). None of the subjects had a personal or familial history of MH. Half of the patients reported one or more prior procedures under general anesthesia, but the anesthetic agents were not reported. Twelve subjects refused IVCT, including 6 from 2008 to 2010, resulting in a study sample of 454 subjects.

From 2008 to 2010, 213 subjects were examined, and 145 of these subjects answered the questionnaire (telephone conversation: n = 118; mailed questionnaire: n = 27). The only difference between the subjects who answered the questionnaire and those who did not was a higher incidence of ARF among those who answered (p = 0.03). The minimum elapsed time from EHS to IVCT was 7 months for these subjects.

### Epidemiological features

Eighty percent of the EHS episodes occurred in metropolitan France, of which 18.1% occurred between November and April. Overseas, EHS occurred in sub-Saharan Africa (n = 35, 42%), French Polynesia (n = 12, 15%), the West Indies (n = 10, 12%), French Guyana (n = 16, 21%), Afghanistan (n = 2, 2%) and Kosovo (n = 7, 8%). The median time from arrival in an overseas country to EHS was 60 days (30–90). As reported previously, the median ambient temperature was 22°C (17–27), and the median hygrometry was 70% (59–79) [15].

The conditions that elicited EHS were available for 407 subjects. EHS mainly occurred during scheduled physical activities (90%): running for 8 km with boots and a combat jacket (61%); running for 8 km with complete battle dress, including an 11-kg rucksack (23%); or regimental cross-country running (6%). All EHS episodes were observed during strenuous exercise.

Among the 145 subjects of the 2008–2010 cohort who answered the questionnaire, the median performance distance of the 12-minute run test (Cooper test), in which subjects were asked to run as far as possible in 12 minutes, was 3000 m (2900–3150) for men and 2700 m (2600–2900) for women. The median time to perform an 8-km run with boots and a combat jacket was 38 minutes (37–40) for men (n = 84) and 41 minutes (39–45) for women (n = 11). The median body mass index (BMI) was 24.6 kg·m^-2^ (22.9–26.3) and was >25 in 41.7% of cases.

### Clinical features

By definition, central nervous system dysfunction was present in all subjects, ranging from confusion (46.0%) to coma (54.0%). Seizures were observed in 3.7% of the patients, with or without coma. Thirty-two patients were admitted to an intensive care unit (ICU). The mean Tco was 40±1°C and exceeded 41°C in 24.0% of cases.

The clinical features of the subjects who were assessed by the questionnaire from 2008–2010 are shown in Table 1.

**Table 1:** Clinical features of exertional heat stroke, 2008–2010 cohort with questionnaire.

Signs and symptoms	Percentage (number)
**Prodromal signs**	
Thirstiness	63.5% (92)
Heavy sweating	60.0% (87)
Exhaustion	59.3% (86)
Disturbed gait	46.2% (67)
Visual field narrowing	31.0% (45)
Vomiting	27.6% (40)
Nausea	22.8% (33)
Headache	18.8% (27)
Lightheadedness	14.5% (21)
Absence of sweating	9.0% (13)
**Central nervous system impairment**	
Coma	60.7% (88)
Confusion	57.9% (84)
Seizures	3.7% (6)

### IVCT status

MH susceptibility was found in 45.6% of subjects. Of them, MHSh was found in 18.5% of subjects, MHSc was found in 9.9% of subjects and MHS for both halothane and caffeine was found in 17.2% of subjects.

Muscle biopsies revealed non-specific morphological abnormalities in 16.7% of cases (mostly type I fiber atrophy) regardless of the MH status (p = 0.56). Five cases demonstrated AMP deaminase deficiency (1 MHS, 2 MHSh and 2 MHN subjects). The cores of two subjects showed no evidence of clinical myopathy (1 MHS subject and 1 MHSh subject).

Table 2 shows the IVCT status according to demographic, clinical and biological data. There were no differences between MHN and MHS patients.

**Table 2:** *In vitro* contracture test status, 2004–2010 cohort.

	*in vitro* contracture test		p-value
	MHN	MHS^a^	
**Number of subjects (%)**	247 (54.4%)	207 (45.6%)	
**Demographic data**			
Age (mean±sd)	25.8±5.2	25.8±5.4	0.96
Sex ratio (M/F)	225/22	199/8	0.04
**Circumstances**			0.58
Cross country	11	13	
Running + jacket	133	111	
Running + load	53	36	
Other	19	19	
**Clinical data**			
Temperature	40.1±0.9°C	40.0±1°C	0.43
Coma	130 (53.1%)	115 (46.9%)	0.81
Seizures	11 (64.7%)	6 (35.3%)	0.68
Confusion	111 (53.1%)	98 (46.9%)	0.61
**Biological data**			
Renal failure	34 (50.7%)	33 (49.3%)	0.90
Hepatic failure	21 (46.7%)	24 (53.3%)	0.56

Legend: MHN, malignant hyperthermia normal response; MHS, malignant hyperthermia susceptible.

^a^Including MHS for caffeine and MHS for halothane patients.

### EHS recurrence

The EHS recurrence rate, as assessed in the 2004–2010 cohort, was 12.7% (n = 59). All recurrent EHS occurred before the muscle investigations. EHS recurrence was not associated with MH status (p = 0.15).

None of the subjects in the 2008–2010 cohort who answered the questionnaire reported EHS after undergoing muscle biopsies, with a median time from the last EHS to the time of the survey of 4 (3–5) years. EHS recurrence occurred prior to the 36^th^ month in 68% of cases. EHS recurrence was not associated with any clinical or biological features, including BMI and physical fitness (Table 3).

**Table 3:** Exertional heat stroke recurrence, 2008–2010 cohort with questionnaire.

	EHS recurrence		p-value
	Yes	No	
**Number of subjects** (%)	14 (9.7%)	131 (89.3%)	
**Demographic data**			
Age (y)	28.3 ± 8.4	25.4 ± 5.2	0.21
Sex ratio (M/F)	11/3	120/11	0.14
BMI	25.7±4.9	24.9±2.8	0.82
BMI > 25 kg.m^-2^	4	56	0.30
Cooper test (m)^a^	2917 ± 343	2940 ± 337	0.82
**Clinical data**			
Coma	9	79	0.77
Seizures	2	4	0.10
Confusion	10	74	0.28
Tco ≥ 41°C	4	27	0.50
**Biological data**			
Renal failure	2	27	0.38
Hepatic insufficiency	0	14	0.10
**Admission to ICU**	1	20	0.69

Legend: BMI, body mass index; EHS, exertional heat stroke; ICU, intensive care unit; Tco, body core temperature.

^a^For male subjects between 20 and 29 years of age, the results are ‘excellent’ for distances > 2800 m, ‘good’ for distances of 2400–2800 m, ‘average’ for distances between 2200 and 2400 m, ‘low’ for distances between 1600 and 2200 m and ‘weak’ for distances < 1600 m [19].

Three subjects had 3 EHS episodes before they were investigated. Their muscle biopsies were normal. Two subjects had MHS, and their EHS episodes occurred within two years. Both belonged to the Foreign Legion, and they were 21 and 24 years old at their first EHS episode; neither exhibited severe EHS. The third subject was a 22-year-old woman experiencing her first EHS episode. She had three EHS episodes within 27 months without severe presentation before a muscle biopsy was performed. Her IVCT status was MHSh.

### IVCT among EHS and MH patients

**Fig 1:**
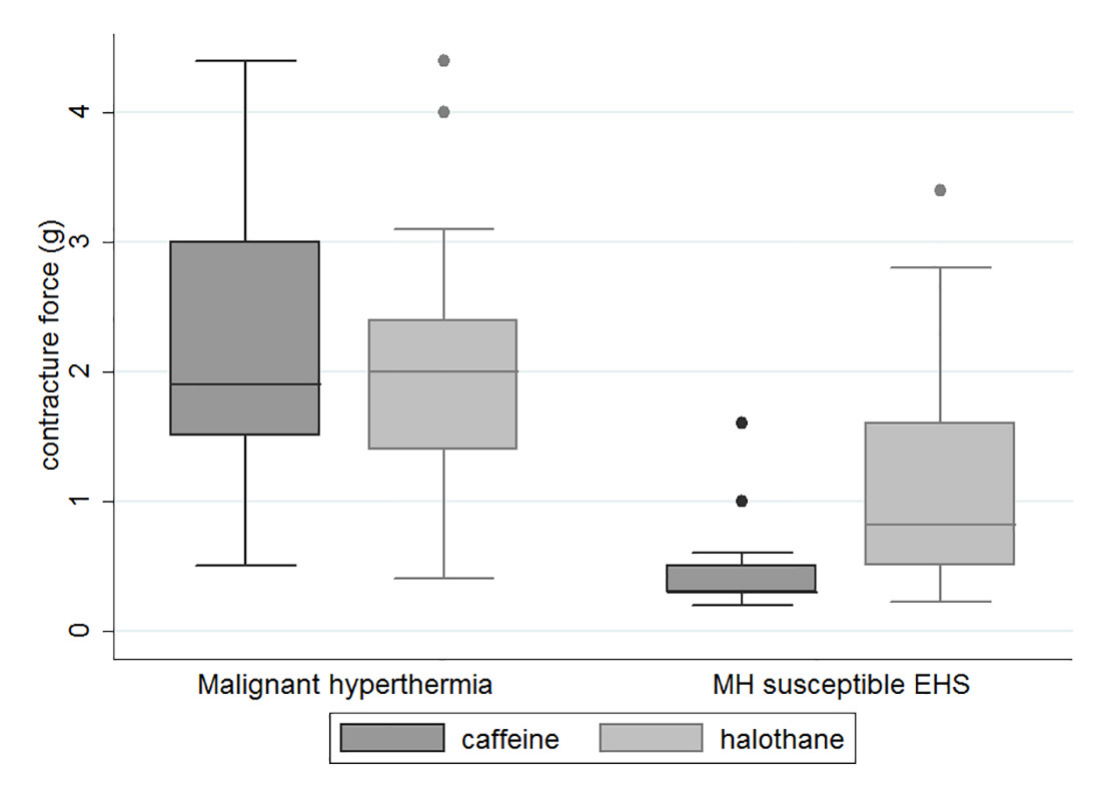
*In vitro* contracture test response for malignant hyperthermia and malignant hyperthermia-susceptible EHS patients. *In vitro* muscle bundle contracture force (g) for halothane and caffeine among malignant hyperthermia patients (left) and among IVCT-diagnosed malignant hyperthermia-susceptible patients with a history of exertional heat stroke (right) (p<0.0001 for halothane and caffeine). Abbreviations: IVCT, *in vitro* contracture test; EHS, exertional heat stroke.

The control group consisted of 34 patients with histories of MH who were investigated between 2003 and 2011 (median age, 27 years (23–32) (p = 0.7); 11 women (p = 0.03)). The median muscle bundle contracture for halothane was 2 g (1.4–2.4) for the MH patients versus 0.82 g (0.5–1.6) for the iMHS EHS patients (p<0.0001) (Fig 1). The median muscle bundle contracture for caffeine was 1.9 g (1.5–3) for the MH patients versus 0.3 g (0.3–0.5) for the iMHS EHS patients (p<0.0001).

## Discussion

This study showed that the clinical and biological features as well as the outcomes of EHS were similar for MHN and MHS patients and that an unexpectedly high prevalence of the MH susceptibility trait (45.6%) was observed among EHS subjects.

Although this study reported on the largest cohort of EHS patients with an IVCT determination to date, the 39°C threshold used in the inclusion criteria and the site of the biopsy used for the determination of IVCT are two methodological issues that should be addressed. First, the 39°C threshold was recommended by the French Armed Forces in an effort to increase sensitivity, taking into account the time-to-temperature assessment due to field conditions and the use of immediate cooling procedures. Because it is generally thought that a Tco >40°C is mandatory for a diagnosis of EHS [20], a lack of specificity might be entertained, as well as conditions other than EHS that may have been included, such as exertional heat exhaustion. Nevertheless, severe CNS impairment, such as confusion, delirium, coma or seizures, was required in all cases. These features are not indicative of exertional heat exhaustion, in which only minor CNS dysfunctions, such as headache or dizziness, are described [20]. Thus, the inclusion criteria were complied with Bouchama’s proposed definition of EHS, in which encephalopathy predominates in the context of hyperthermia but without a formal threshold [6]. Second, the biopsy was performed in the *biceps brachialis* muscle rather than in the *vastus lateralis* muscle because the recovery of the *biceps brachialis* muscle function was more rapid. Both muscles are proximal with similar fiber-type compositions, and MH is a genetic disorder with clinical features that are not restricted to the lower limbs. Bendahan et al. provided evidence that IVCT results did not differ when the biopsy was excised from the *biceps brachialis* rather than the *vastus lateralis* [18].

In the present study, patients with exertional rhabdomyolysis were not included for the following reasons. First, rhabdomyolysis physiologically occurs after exercise, especially eccentric exercise. Clarkson et al. reported CPK values >10,000 U/l at four days after 50 eccentric contractions of the elbow flexor muscles without renal dysfunction in 25% of cases [21]. Second, the maximum CPK levels were not associated with EHS severity in our cohort [22]. Third, the diagnosis of rhabdomyolysis relies on biological and not clinical criteria, *i*.*e*., signs such as cramps, weakness or effort-induced pain, which occur during normal physical exhaustion [23]. Although some myopathies are associated with rhabdomyolysis, the muscle biopsy was normal in the majority of the cases in our cohort. The clinical delineation of rhabdomyolysis is not as stringent as that of EHS.

Unlike previously published studies, EHS was not associated with a low level of fitness. In this study, the subjects were physically fit, considering that the median 12-minute run test (Cooper test) was 3000 m. In the Cooper test (developed in the late 1960s), which is a field-test estimate of maximum oxygen consumption, a running distance greater than 2800 m is ranked as “excellent” [19]. Previous studies have reported an inverse association that linked poor physical fitness and the risk of developing EHS [24,25]. The physical differences between US and French soldiers may also account for this discrepancy [26].

The key finding of this study was the unexpectedly high prevalence of the MH susceptibility trait (45.6% or 17.2% when restricted to MH susceptible subjects for both halothane and caffeine) among EHS subjects. This prevalence is much higher than the 1.48% frequency reported in asymptomatic patients by Ording et al. and much higher that of the French population (*i*.*e*. approximately 1/2,000 to 1/3,000), although these studies assessed MH susceptibility when subjects had abnormal results for both halothane and caffeine [27,28]. At first glance, this result supports the hypothesis that there is a common sub-clinical myopathy linking the two conditions, with MH being considered as a risk factor of EHS. Although it is not possible to rule out this hypothesis, this straightforward conclusion may be hampered by the following arguments. First, the MH susceptibility status was ascertained after the onset of EHS, whereas it should be ascertained before EHS to retain the causal role of MH. Second, the existing literature reporting EHS after MH consists of a single case report [29]; furthermore, MH susceptibility after EHS was reported in small case series before this study [9,10,14,30]. Another study showed that 10 of 12 post-exertional rhabdomyolysis patients exhibited MH susceptibility [31]. However, rhabdomyolysis alone is not indicative of EHS, and the sole patient who fulfilled the clinical criteria for EHS was MHSh. Third, the IVCT test is not specific to MH, and a positive IVCT is observed in some myopathies such as Duchenne dystrophy and central core disease [32]. Fourth, the IVCT responses of iMHS EHS patients were much less critical than those of MH patients in our study; however, the reason for this finding is unclear. The iMHS EHS patients could harbor an infra-clinic channel dysfunction. This would account for their less efficient calcium homeostasis, as evidenced by a positive IVCT. Furthermore, disturbances in calcium homeostasis and muscle energy production may coexist in EHS subjects with the MH trait [33]. A disturbance in muscle energetics has been demonstrated in MHS subjects using ^31^P magnetic resonance spectroscopy [34,35]. This disturbance in calcium homeostasis could also result in increased caloric production among iMHS EHS patients [36]. Fifth, whole genome sequencing of RYR1 identified more variants among MHN patients than MHS patients in a cohort of 28 subjects with history of exertional heat illness; however, the clinical relevance of this finding remains unclear [37].

In conclusion, this study investigated MH susceptibility among EHS patients; however, the questions of whether EHS patients had an increased risk of MH and whether MH susceptibility was a risk factor for EHS could not be answered.

A return to play or duty for athletes or members of the military, respectively, is a crucial issue after an EHS episode [38]. Importantly, markers of recurrence should be identified, as such markers may help clinicians distinguish subjects with a high risk of subsequent EHS from subjects with a low risk of recurrence. In the present study, the MH susceptibility status was not useful as a marker of recurrence; however, its diagnostic counterpart, a positive IVCT, could serve as a surrogate marker of subclinical myopathies.
